# Cell body shape and directional movement stability in human-induced pluripotent stem cell-derived dopaminergic neurons

**DOI:** 10.1038/s41598-020-62598-4

**Published:** 2020-04-02

**Authors:** Yuko Arioka, Emiko Shishido, Itaru Kushima, Daisuke Mori, Norio Ozaki

**Affiliations:** 1 Department of Psychiatry, Nagoya University Graduate School of Medicine, Nagoya, Japan; 2 Center for Advanced Medicine and Clinical Research, Nagoya University Hospital, Nagoya, Japan; 3 Institute for Advanced Research, Nagoya University, Nagoya, Japan; 4 National Institute for Physiological Sciences, Okazaki, Japan; 5 Medical Genomics Center, Nagoya University Hospital, Nagoya, Japan; 6 Brain and Mind Research Center, Nagoya University, Nagoya, Japan

**Keywords:** Cellular motility, Cell migration, Cellular neuroscience

## Abstract

Neuronal migration is necessary in the process of the formation of brain architecture. Recently, we demonstrated that human induced pluripotent stem cell (iPSC)-derived dopaminergic neurons exhibit directional migration *in vitro*. However, it remains unclear how the cell shape is involved in their migration. In this study, we performed live imaging analyses using human iPSC-derived dopaminergic neurons. Our automated method, which can automatically identify the cell body shape and the cell position at specific time points, revealed that healthy iPSC-derived dopaminergic neurons migrate according to their shape. This migration behavior was out of accord in neurons derived from iPSCs carrying an *RELN* deletion. Our findings provide a novel theory that cell body orientation is related to the stability of movement direction for human dopaminergic neurons, under the regulation of *RELN*.

## Introduction

Neuronal migration is essential for the correct development of the nervous system. During mammalian brain development, nascent neurons migrate from their birthplace to their designated location via a strictly controlled process that enables the formation of complex neuronal architectures^1^. Live imaging technology using rodents revealed that various controlled dynamic phenomena occur during neuronal migration, including time-dependent alterations^2,3^. Therefore, maintaining the correct migration behavior is necessary for the formation of an anatomically accurate brain.

Although understanding neuronal migration in rodents has rapidly increased in recent years, the neurodevelopmental events and dynamic phenomena that occur in humans remain largely unclear, partly due to ethical concerns involving human brain studies. Because of species differences in neuroanatomy, a better understanding of the dynamics involved in human brain development is required to elucidate brain function and provide insight into human brain diseases. Recent studies involving human-induced pluripotent stem cell (iPSC)-derived neurons revealed their potential as a powerful tool for studying human neuronal development^4^. iPSC-derived neurons will provide novel information regarding neuronal dynamics during human brain development.

Recently, our single-cell trajectory analysis using time-lapse images of migrating neurons revealed that human iPSC-derived dopaminergic neurons exhibit directional migration, which was weakened in dopaminergic neurons carrying a rare *RELN* variant (RELN-del)^5^. The direction of cell movement is reportedly controlled by the patterns of cell shape in *Dictyostelium* cells^6^. Thus, here the novel question is whether the cell shape is associated to neuronal migration in human iPSC-derived dopaminergic neurons.

The present study observed and analyzed the migration behavior of human iPSC-derived dopaminergic neurons using an automated detection system, which can identify the cell body shape and the cell position at specific time points. Observation of migrating neurons from healthy controls (HC) revealed that the cell body axes of neurons were aligned with stable movement direction. Conversely, the cell body axes were out of alignment in RELN-del cells. Therefore, migration stability is associated with the robustness of the cell axis rotation, and *RELN* plays an important role in this process.

## Results

### Cell shape differs in RELN-del dopaminergic neurons

**Figure 1:**
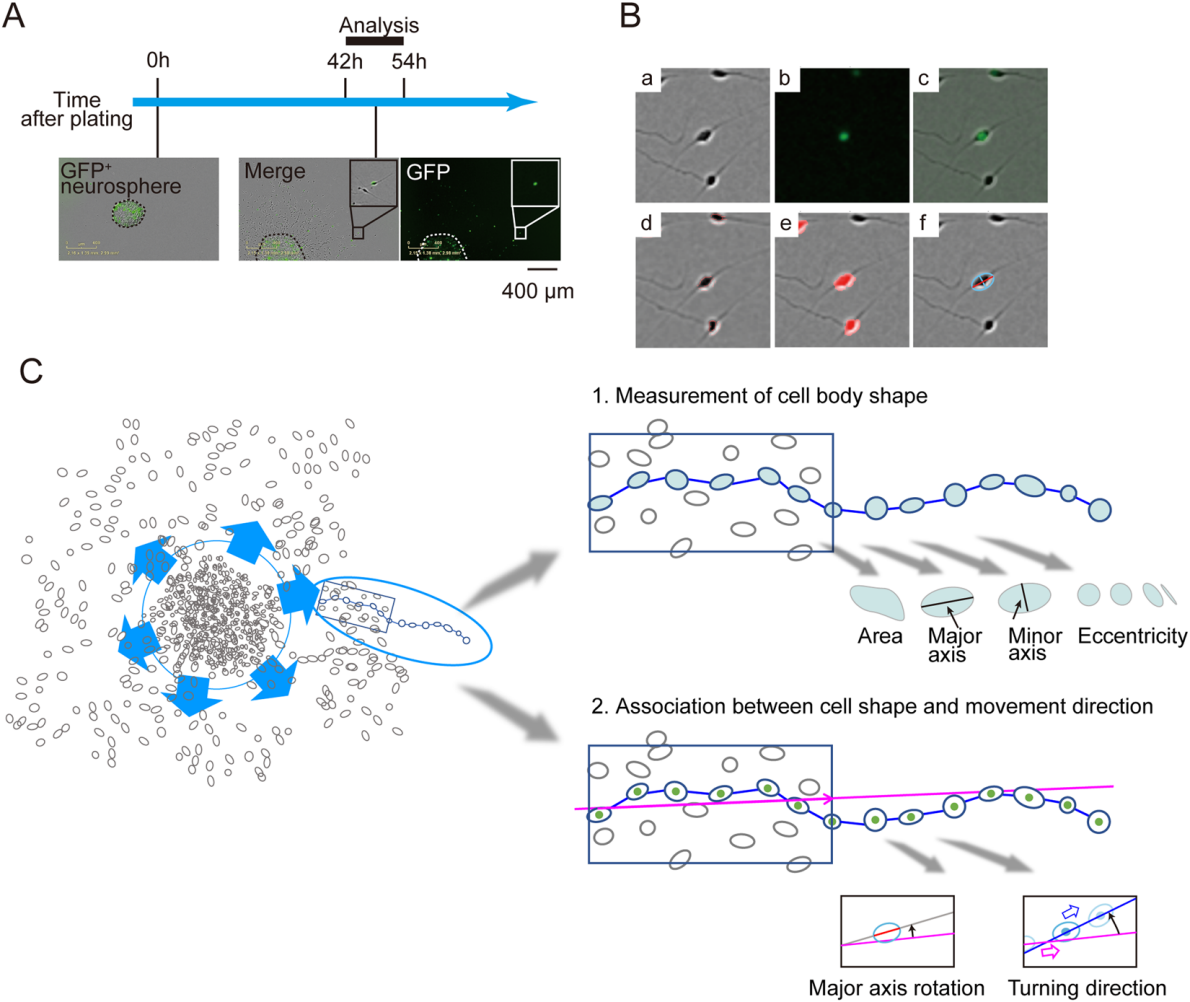
Automated method for identifying cell shape and position. **(A)** Experimental scheme. Cell aggregates (neurosphere) containing GFP-positive (GFP^+^) cells were plated onto Matrigel-coated dishes. Images of the GFP and phase-contrast optics from 42 to 54 h were analyzed. Dashed lines represent the edges of the aggregates. Insets show magnified images of the cells that migrated out from the aggregates. **(B)** Representative example of the automated detection of the cell body area. Images in **(**a–f**)** are from the same frame taken at 42 h. (a) Phase-contrast image. (b) Nuclear GFP signal. (c) Merged image of the phase-contrast image and GFP signal. (d) Edge detection using a sobel filter (red). (e) Selected area of a cell body (red). (f) Fitted ellipse and its minor axis of the central cell (pale blue) and the major axis (red). (**C**) Analysis strategy of cell shape and migration behaviors. We captured the time lapse images of migrating neurons *in vitro* and tracked a single cell by GFP signal (*Left*). In step 1 (*Right upper*), from the video image of migrating cells, we measured the size of cell-body areas, major/minor axis and eccentricity of the fitted ellipses. These features related to cell-shape were then compared between HC and RELN-del cells (Fig. 2). In step 2 (*Right lower*), we calculated the projected path (pink line) of each cell by using the first half of positional data. Using the projected path as the reference path, we created an evaluation method to test if the major axis rotation was associated with the movement direction. Then, we compared between HC and RELN-del cells (Figs. 3–5).

Highly homogenous (>85%) tyrosine hydroxylase (TH)-positive neurons were differentiated from HC iPSCs (Suppl. Fig. 1A,B). Similarly, RELN-del iPSCs [both of RELN-del patient (PA) and RELN-del isogenic] highly differentiated into TH-positive neurons (>85%) as reported in our previous study^5^. After plating neurospheres, neurons extended from the place of aggregation (Fig. 1A). Nuclei were labeled using green fluorescent protein (GFP) to trace migrating neurons (Fig. 1A). Images were captured at 15 min intervals 42–54 h after plating, and these time-lapse images were analyzed. We established an automated method to identify the region of cell bodies of GFP-positive neurons (Fig. 1B, Suppl. Movies 1 and 2) using image processing (see Experimental procedures). Briefly, the cell body area in each video frame was outlined and segmented from the phase-contrast images (Fig. 1Ba–e). The cell body region was then fitted into an ellipse (Fig. 1Bf). Using this, we analyzed the following two points: 1) Measurement of cell body shape and 2) Association between cell shape and movement direction (Fig. 1C).

**Figure 2:**
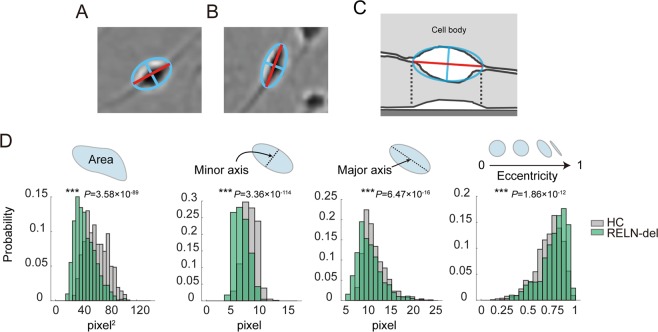
Cell shape analysis of iPSC-derived dopaminergic neurons. Representative images of the cell body region of HC **(A)** and RELN-del **(B)** cells. The fitted ellipse and its minor axis of the central cell are shown in blue, and the major axis is shown in red. **(C)** Schematic illustration showing the horizontal (top) and vertical (bottom) plane images of the cell. The fitted ellipse and its minor axis of the central cell are shown in blue, and the major axis is shown in red. **(D)** Comparison between the area, length of the minor/major axes of a cell body, and eccentricity of the fitted ellipse of a cell body of HC (gray) and RELN-del (green) cells. ***P < 0.001 (HC cells vs RELN-del cells, Associated with Table 1).

First, we calculated the length of the minor/major axes and eccentricity of the cell body, using the fitted ellipse (Fig. 2A–C). We compared the cell body shape between HC and RELN-del cells. The numerical distribution of the area size of the cell bodies, length of minor and major axes, and eccentricity of the cell body of RELN-del cells were significantly different from that of HC cells (Table 1, Fig. 2D and Suppl. Fig. 2). These results revealed differences in the shape of RELN-del and HC cell bodies.

**Table 1:** Numerical difference between HC and RELN-del cells.

	HC n = 785	RELN-del n = 1032	*P-*value
Area (pixel^2^)	60.6 ± 16.8	44.1 ± 15.6	3.58 × 10^−89^
Minor axis (pixel)	7.22 ± 1.14	5.87 ± 1.19	3.36 × 10^−114^
Major axis (pixel)	11.2 ± 2.48	10.2 ± 2.78	6.47 × 10^−16^
Eccentricity	0.719 ± 0.143	0.767 ± 0.145	1.86 × 10^−12^

We investigated the accuracy of this methodology as this was a new method that uses image processing. Several different validation methods were used to confirm the accuracy of the analysis (see Experimental procedures). One indicator was the area of the region that was extracted by this method. The average pixel area of the extracted region of the cell body in HC cells and RELN-del cells were similar to those measured by hand (57.8 ± 8.7 pixels, n = 240; and 41.2 ± 8.6 pixels, n = 300, respectively), confirming that our automated detection of the cell body region was comparable with manual detection. In addition, there is another concern; the results regarding cell shape may vary from the different passage numbers after iPSC establishment. To address this, we compared the results between HC1 cells at different passage numbers. Although some differences were detected (Suppl. Fig. 3), they were smaller than those in comparison between HC cells and RELN-del cells. For example, the value of major axis was not altered even if cells were from the different passaged iPSCs.

### Excessive cell body rotation in RELN-del dopaminergic neurons

We found a significant difference in cell body shape between HC cells and RELN-del cells. Since RELN-del dopaminergic neurons showed less directionality compared with HC cells during migration^5^, we speculated the association of cell body shape with directionality. Therefore, we investigated the rotation behavior of the cell body using three steps.

**Figure 3:**
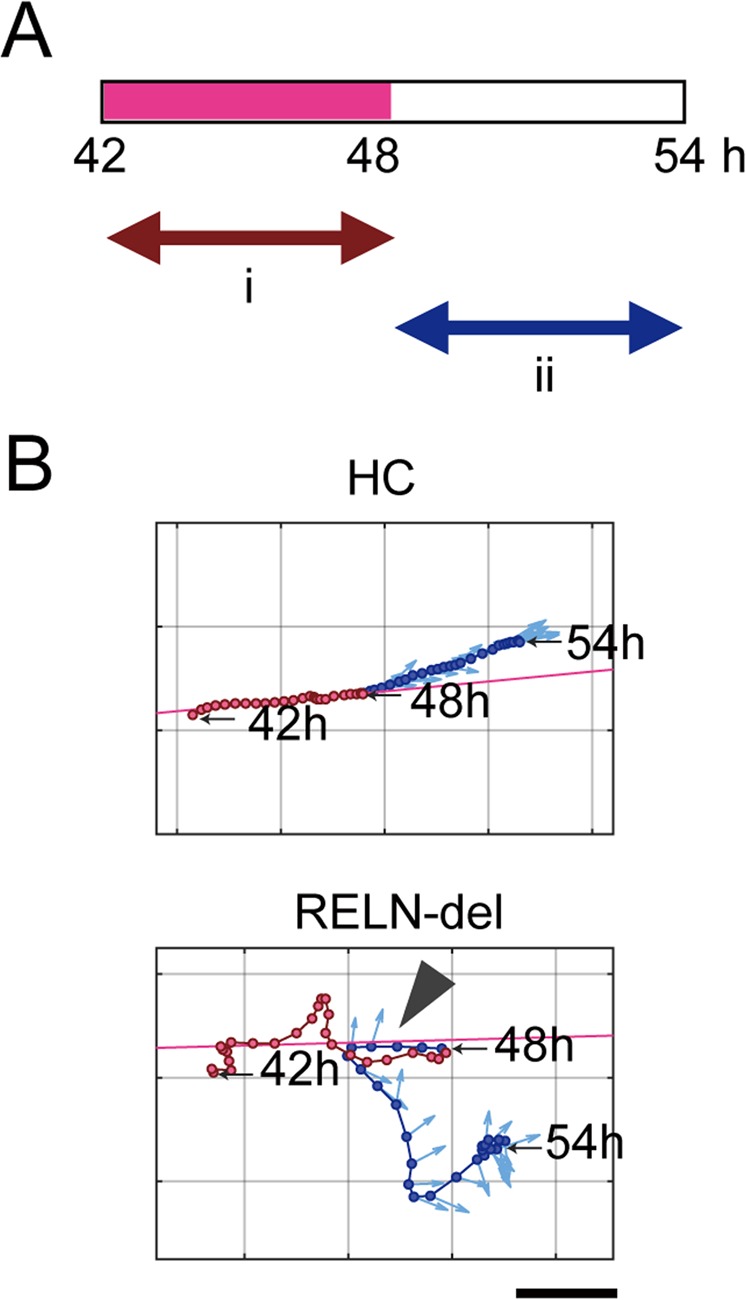
Time-lapse analysis of migrating iPSC-derived dopaminergic neurons. **(A)** Scheme of time-lapse analysis. Positional data of cells (GFP) in the first half (i) (pink interval in the time bar) was used to define the projected path. **(B)** Examples of the trajectories of HC and RELN-del cells. The pink line in each plot denotes the projected path. The red circles indicate cells at 42–47.75 h (i), whereas blue circles indicate cells at 48–54 h (ii). Pale blue arrows denote the cell major axis orientation. Arrowhead indicates the point at which the cell turned to the opposite direction. Time points at 42, 48, and 54 h are noted. Scale bar represents 60 μm.

**Figure 4:**
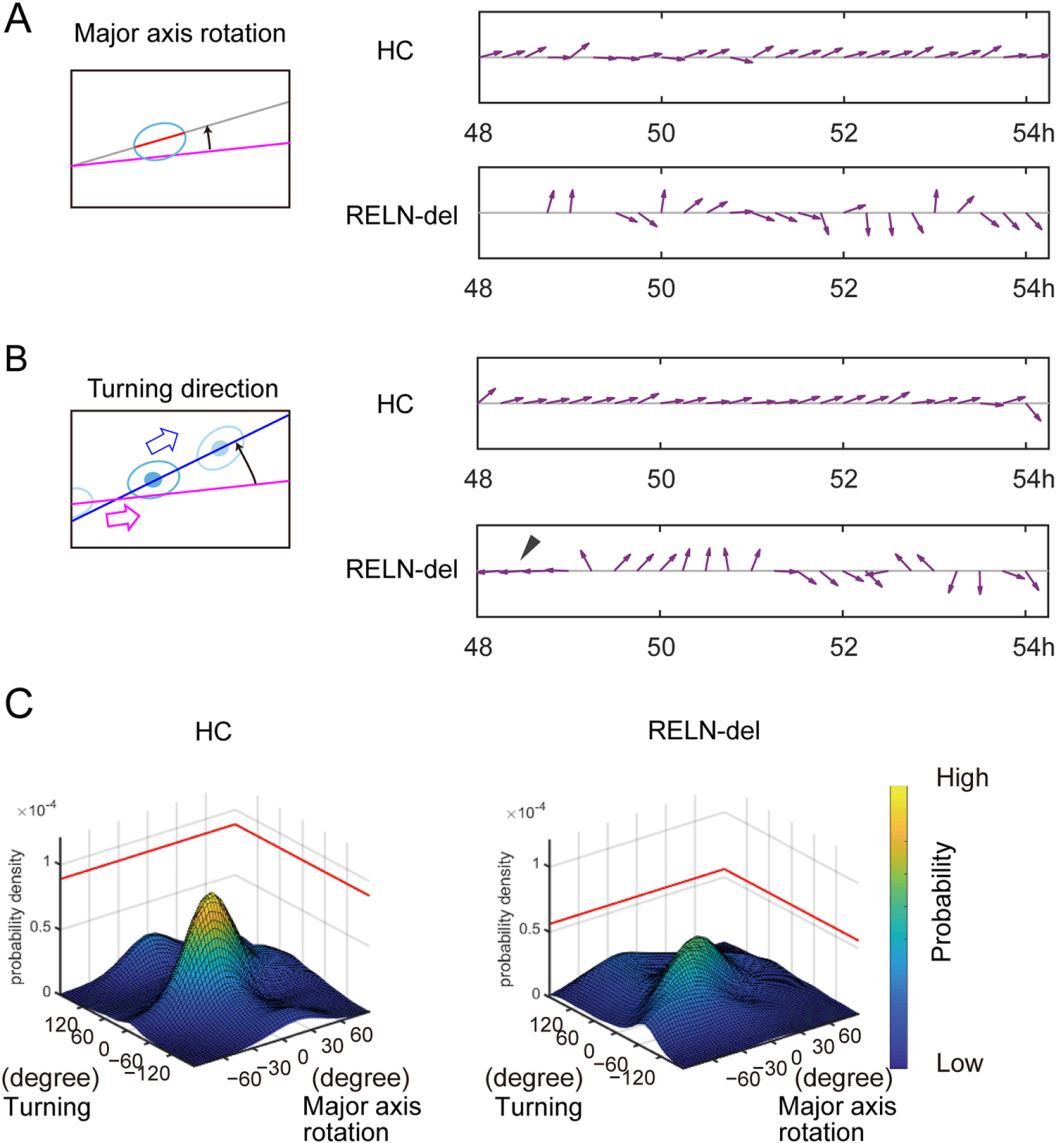
Cell axis rotation and turning direction in HC and RELN-del cells. **(A)** *Left*: Illustration to show the rotational angle of the major axis (red segmented line) to the projected path (pink line). *Right*: Representative quiver plots of the major axis rotation in HC and RELN-del cells. The inclination of the arrows indicates the rotational angle to the projected line. The cells shown for each named group are the same cells shown in Fig. 3B. **(B)** *Left*: Illustration to show the turning angle of the movement vector (blue line) to the projected path (pink line). *Right*: Representative quiver plots of turning angle in HC cells and in RELN-del cells. The arrowhead indicates the same time point as the arrowhead in Fig. 3B. The cells shown for each named group are the same cells as those shown in (Fig. 3B). **(C)** Probability distribution of the bivariate angular data in (A) and (B). The peak probability density in RELN-del cells was significantly lower than that in HC cells (*P* < 0.0001, 0 in 10,000 trials by random sampling test, associated with Suppl. Fig. 4A).

In the first step, we examined the angular rotation of the cell body on the projected path of migration. We divided the position data of each cell into two parts: the first half (42–47.75 h) and the latter half (48–52 h) after plating neurospheres (Fig. 3A). The projected path was determined using regression analysis of data from the first half and was set as the reference direction the cells were expected to follow (pink lines in Fig. 3B). Then, we examined the rotation of the major axis on the projected path in the latter half of the time course (Fig. 4A, *Left*). The rotational angle of HC cells was almost consistent with the projected path, whereas the cell axes of RELN-del cells were frequently different from the projected path (Fig. 4A, *Right*).

In the second step, we examined the turning direction of cell movement on the projected path (Fig. 4B, *Left*). The turning angle to the projected path was measured for each video frame at 15 min intervals. The turning angle of HC cells was small, indicating that the cells followed the expected direction. In contrast, RELN-del cells moved away from the projected path and occasionally moved in the opposite direction (arrowhead in Fig. 4B, *Right*).

To obtain a general overview, we compared the probability distribution between the above two variables. Both HC cells and RELN-del cells exhibited a unimodal distribution with prominent peaks at the center (Fig. 4C), indicating that the rotation and movement direction of cells tended to follow the projected path. However, the peak probability density of RELN-del cells (5.2 × 10^−5^ per degree square) was significantly lower than that of HC cells (8.9 × 10^−5^, 0 in 10000 trials, *P* < 0.0001, Suppl. Fig. 4A). We also analyzed the peak probability density of the HC1 with different passage numbers, confirming that HC cells show high probability density regardless of passage numbers (1.3 × 10^−4^ and 9.0 × 10^−5^, Suppl. Fig. 5A,B). Furthermore, both of RELN-del PA and RELN-del isogenic cells represented a low density (5.7 × 10^−5^ and 5.6 × 10^−5^ respectively, Suppl. Fig. 5C,D). HC cell movements were predictable according to major axes of their cell bodies and past movement directions, whereas RELN-dell cell movements were not.

**Figure 5:**
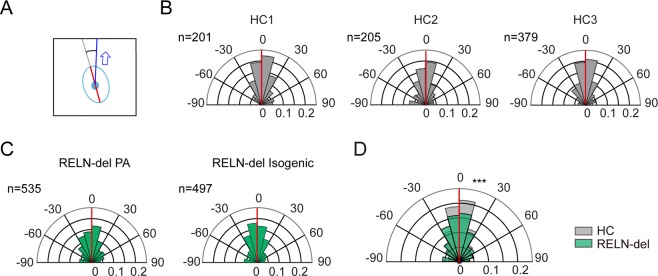
Accordance rate of the movement vector to the cell major axis. **(A)** Schematic representation showing the directional difference between the movement vector (blue arrow) and the cell major axis (red segmented line). **(B)** Distribution of the directional difference in HC cells. The angular distribution (degree) was shown in polar plots, and the spokes denote the probability in each population. The three plots show the distribution from three different HC cell lines. **(C)** Distribution of the directional difference of the movement vector in RELN-del cell lines. **(D)** The results from the HC and RELN-del cell lines were combined and overlaid. The difference between the two groups was statistically significant (***P < 0.001, 0 in 1000 trial by random sampling test, associated with Suppl. Fig. 4B). The numbers (n) indicated in each plot represent number of image frames used for the analysis.

The mismatch of RELN-del cell behavior to that of the projected path may have been due to a lack of coordination between cell movement direction and major axis orientation. Thus, in the third analysis step, we examined whether the cell major axis differed from the cell movement direction (Fig. 5A). As shown in Fig. 5B–D, the orientation of the major axis was associated with the direction of movement of both HC and RELN-del cells. However, focusing on the angular difference from −15° to 15°, HC cells showed 39.6% of total instances, whereas this dropped to 32.8% for RELN-del cells. It was significantly different between the two cell types (0 in 1000 trials, *P* < 0.001, Suppl. Fig. 4B).

## Discussion

**Figure 6:**
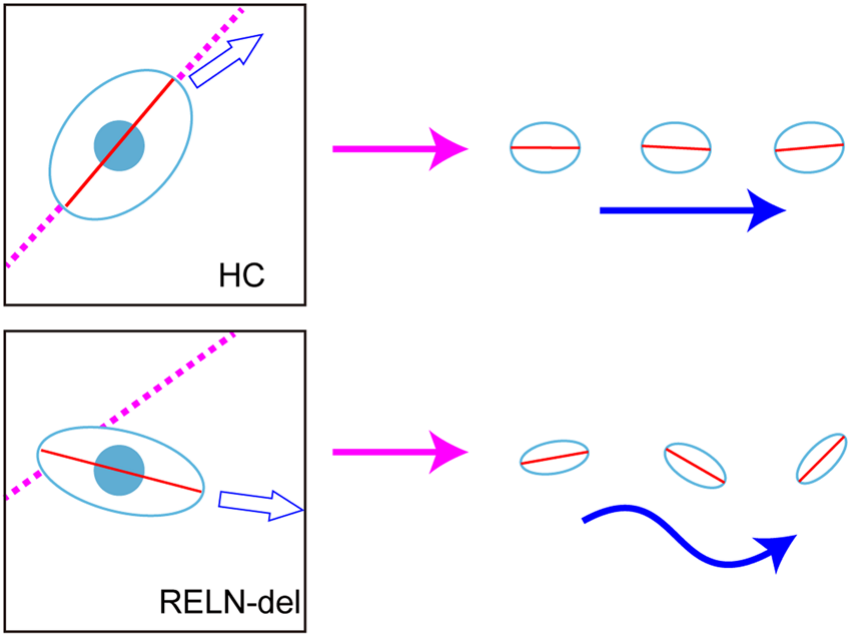
Graphical abstract of the migratory behavior of HC and RELN-del cells. *Left*: In HC cells, the cell axes (red segmented lines) coordinated with the projected path (pink dashed lines), and the movement direction (blue hollow arrow) was consistent with the projected path. In contrast, in RELN-del cells, the cell axes and movement direction were poorly coordinated with the projected path. *Right*: In HC cells, the major axis (red segmented line) followed the projected path (pink arrow), making a stable trajectory in the latter half of movement (blue arrow); however, this tendency was disrupted in RELN-del cells with different cell shape, resulting in unstable migration.

We examined cell body shape in migrating human iPSC-derived dopaminergic neurons and revealed differences between RELN-del and HC cells. To our knowledge, this is the first study to report differences in cell body shape between HC and RELN-del dopaminergic neurons in humans (Fig. 6). We also showed shape-associated migration in HC cells. The major axis of HC cells followed the projected path, making a stable trajectory in the latter half of movement; however, this tendency was weak in RELN-del cells (Fig. 6). These findings support our previous study of the wondering pattern of migration in RELN-del cells^5^. Although the mechanisms of cell migration are not fully understood, reelin protein is known to play an important role in this process^7^. Our results suggest a novel theory that cell body orientation is related to the stability of movement direction.

In the present study, migrating neurons continued to move in an outward direction and away from the original position of cell aggregates (neurospheres) where neurons were born. Our findings prompted us to investigate (1) the shape of the cell body; (2) the connection between the cell movement machinery and cell body shape; (3) the role of reelin in this process; and (4) how neurons maintain a stable outward movement in the neighboring neurons in the absence of a positional cue for direction.

First, we propose that the cytoskeleton plays an important role in both cell shape and movement during iPSC-derived dopaminergic neurons. Actin filaments are central players in both cell shape and movement^8^. A model of actin network treadmilling can quantitatively predict cell shape^9^. Therefore, the tension formed by these molecules also may be a major contributor of cell body shape in human iPSC-derived dopaminergic neurons.

Second, the present study revealed a correlation between the cell axis and movement direction in human neurons. A shape-associated migratory direction as observed in our analysis is a reasonable migration mechanism for neurons. The migration of cells adhered to a two-dimensional substrate consists of cycles of cell local attachment and detachment. Matrigel includes extracellular matrix components, such as laminine and collagen IV, which promote cell attachment and regulate cell shapes and migration patterns in brains^10^. Therefore, our findings indicate extracellular matrix-dependent relationships between cell shape and migration. Moreover, because RELN-del cells had decreased cell-body areas, their contact with Matrigel may be reduced, leading to loss of shape-associated migratory directions.

Third, reelin is a protein encoded by the *RELN* gene that is known to control neuronal migration^7^. We revealed that the coordination between the cell axis and movement direction was lost in dopaminergic neurons derived from patients and isogenic iPSCs carrying RELN-del. We also showed that the eccentricity of RELN-del cells was greater than that of HC cells. As previously reported, reelin deficiency leads to polarity defects with destabilization of the actin cytoskeleton in cortical neurons^11,12^. The findings of the present study may reflect disruption of the polarity and/or destabilization of actin cytoskeleton in the cell body due to RELN-del. Moreover, a recent study using mice reported that reelin stabilizes the leading process morphology in dopaminergic neurons during their migration^13^. Thus, reelin may control neuronal migration via regulation of whole neuronal morphology, including the cell body and associated processes, even *in vitro*, such as in human iPSC-derived dopaminergic neurons. Further studies using human iPSC are required to clarify the involvement of reelin in neuronal migration.

Last, neuronal migration is a stable and outward movement; however, it is unclear how neurons know their relative position in terms of migrating neighbors. Furthermore, attractive/repulsive molecules such as netrin were not assumed in our system. The neurons migrate from an aggregate to a vacant place, so it is difficult to assume that external chemotropic molecules lead the migration. It is possible that once neurons acquire outward directionality while exiting the aggregate, the neurons continue to be stably aligned in the direction of the cell body axis. Supporting this hypothesis, HC cells inductively followed the projected path predicted from past movement, and cell axes were in accordance with the projected path (Figs. 3 and 4). This is the simplest model to explain the different stability of migration between HC and RELN-del cells. This concept may explain the basic mechanisms of the migration system of neurons; although, we do not exclude other possibilities to explain the phenomenon.

In conclusion, we examined the dynamic alterations in cell position and shape and revealed that human iPSC-derived dopaminergic neurons migrate in a shape-associated manner that is dependent on reelin. Our findings may provide novel insights into the mechanisms controlling neuronal migration in humans and demonstrate the usefulness of iPSCs as a model for studying cellular dynamics critical to human neuronal development.

## Methods

### Ethics statement

The generation and application of human iPSCs were approved by the Nagoya University Ethics Committee (approval number: 2012–0184). Written informed consent was obtained from all study participants. All methods were performed in accordance with the relevant guidelines/regulations.

### iPS cells and neuronal differentiation

Human iPSC lines were derived from three HCs [HC1, a 37-year-old Caucasian female 201B7 (HPS0063)^14^; HC2, a 30-year-old Japanese female^15^; and HC3, a 65-year-old Japanese female] and one patient with schizophrenia carrying a rare *RELN* variant (PA, a 58-year-old Japanese male)^5^. Isogenic iPSCs carrying a homozygous *RELN* variant derived from HC1^5^ were also used. HC1-derived iPSC lines were provided by RIKEN BRC (Japan), and the other iPSC lines were generated from peripheral blood monocytes using episomal vectors. The criteria for iPSCs were as follows: (1) expressing pluripotent stem cell markers (TRA-1-60 and NANOG); (2) able to differentiate into three germ layers *in vitro*; and (3) showing no clinical significant copy number variation^16^ using array comparative genomic hybridization. The data of HC3 was described in Suppl. Fig. 6.

Neuronal differentiation was performed as previously reported^5^. Briefly, neurospheres were induced from iPSCs pretreated with SB431542, CHIR99021, and dorsomorphin. Differentiation into neurons was achieved by plating secondary neurospheres onto Matrigel-coated culture dishes. We used 12-well plates coated with ×100-diluted Matrigel (Corning). Cells were cultured with 1 ml medium in an atmosphere containing 5% CO_2_ and 18–22% O_2_.

### Immunocytochemistry

Cells were fixed with 4% paraformaldehyde for 15 min, permeabilized, blocked in phosphate-buffered saline (PBS) containing 0.3% Triton X-100 and 1% bovine serum albumin for 60 min, and incubated with primary antibodies [anti-TH (Millipore), anti-βIII-tubulin (Sigma), anti-TRA-1-60 (abcam), anti-NANOG (abcam), anti-SOX17 (R&D systems) and anti-αSMA (Sigma)] overnight at 4 °C. After washing with PBS, immunolabeled cells were incubated with Alexa488 or 594-tagged secondary antibodies for 1 h at room temperature. Images were captured using a BZ-9000 fluorescence microscope (Keyence, Japan).

### Single-cell migration analysis

We plated secondary neurospheres onto Matrigel-coated culture plates. Cells were labeled with GFP using CellLight Nucleus-GFP (Thermo Fisher, USA). Forty-two hour after plating, time-lapse videos with 15 min intervals were acquired using IncuCyte (Essen Biosciences, USA)^5^. Nuclei were identified by sparse GFP signals in x-y coordinates of the video frame to understand the positioning of the cells. Raw positional data were processed with a smooth filter of five time points. The movement vector obtained a differentiation of the positional data from adjacent frames.

From the time-lapse videos above, both phase-contrast and fluorescent (GFP) images were obtained at each time points, followed by the further analysis in Fig. 1C. For the image analysis, Image Processing Toolbox with MATLAB (The MathWorks, Inc., Natick, U.S.A.) was used. To make the analysis easier, raw image frames were cutout to the size of 244 × 244 pixels with 256 gradations (Fig. 1Ba,b). The phase-contrast images (Fig. 1Ba) were subsequently used to identify the shapes of cell, and the fluorescent images (Fig. 1Bb) were used to identify the position and trajectory of cells. For profiling the shape of cells, several steps of image processing were applied as follows. A sobel filter was used to detect the edges of cell outlines from the grayish images of resized phase-contrast images (Fig. 1Bd). Outlines were dilated and eroded to leave a single image object covering the entire cell body (Fig. 1Be). The image object was then converted to an ellipse with the same normalized second central moments of the object (Fig. 1Bf). Eccentricity was calculated as the ratio of the distance between the foci of the ellipse and its major axis length. Eccentricity indicates that the cell body is not fitted as a perfect circle but elongation in one direction. Depending on the shape of the ellipse, eccentricity varied from a perfect circle (0) to a line segment (1).

The method above made it possible to detect the cell-body region of a single cell; however, in some cases, a misidentification was observed (Suppl Fig. 7). In order to exclude the misidentified cases for further analysis, two methods were used. First, we obtained the convex hull of the image object and calculated the proportion of the pixels of the object in the convex hull. A small proportion indicated that the object was crescent shaped and may not cover the entire region of the cell body. We set the threshold of the proportion at 0.7 and excluded objects with a proportion below this threshold value from the analysis. Second, we checked all the objects and raw images by eye and identified that some had aberrant shapes in the object. Using these two exclusion methods, 60 out of 1639 (3.7%) frames were excluded from data analysis. Finally, to verify the identification method of the cell body region, the cell body area was determined by eye and compared with the automated results.

There still remain some limitations of the image analysis. For example, closely located-two cells are sometimes identified as a single cell (Suppl Fig. 7C,D). Such cases were excluded from the analysis; however, if the cell density affects the ratio of exclusion, it can cause a bias to data interpretation in the comparison between different specimens (e.g., HC vs. RELN-del). To avoid this effect, in addition to the criteria using convex hull (Supple Fig. 7), we selected cells to analyze between 500 and 1000 μm from the edge of aggregation.

### Estimation of movement direction and probability distribution of angular dispersion

We obtained an image video that contains one entire neurosphere and the migrating cells from it. The size of the image video was 1088 × 760 pixels. Next, we detected the nuclei of cells in an x-y coordinate by GFP signal, and traced them with the Cell-Tracking application in ImageJ^5^. To obtain the projected path from the first half of the measurement (between 42 and 47.75 h after plating), we obtained the positional data of cells on x-y coordinate and fitted it to the linear regression model with a robust regression method with bisquare weights (Fig. 3). The movement vector was obtained as the difference of a single cell position between image frames, using a smoothing filter with a window of 5 time-points. The turning angle was calculated as the angular difference between the movement vector and the projected path for each time point (Fig. 4B). The rotation of the cell axis was calculated as the angular difference between the cell major axis and the projected path for each time point (Fig. 4A). Each group of bivariate data, including one from HC cells and one from RELN-del cells, was plotted on a two-dimensional plane, and then the probability distribution was estimated using the kernel smoothing function with a bandwidth of 20°.

### Statistical analysis

Due to the limitation of image analysis, GFP-positive cells that sparsely migrated from the place that we plated neurospheres were subjects for analysis. As a result, the numbers of cells that met these criteria were HC1: n = 7, HC2: n = 6, HC3: n = 11, RELN-del PA: n = 20, and RELN-del isogenic: n = 24. In the image analyzes of cell shape, nuclei were traced with GFP labeling. Time-lapse images from different iPSC lines were pooled [HC1 (n = 7), HC2 (n = 6), and HC3 (n = 11)], and a total of 785 images of HC cells were used for analysis. For RELN-del cells, data from two RELN-del cell lines were pooled [RELN-del PA (n = 20) and RELN-del isogenic (n = 24)], and a total of 1032 images were used for analysis. Two means of the area, length of minor/major axes, and eccentricity of the cell body were compared using Student’s or Welch’s *t* test (unpaired and two-tailed). Unless otherwise stated, *P*-values <0.05 were considered statistically significant.

A random sampling test for the probability distribution of turning angle and major axis rotation, resampling was performed 10,000 times using HC data as a seed to determine whether the peak probability density reached the level of the RELN-del peak probability density. A random sampling test for the angular difference of cell major axis and movement vector was performed as follows. Randomly selected and appropriately scaled datasets from HC cells were pooled. Then, the proportion of the subgroup with ±15° to the whole population was used to verify whether the two groups had the same probability distribution. The resampling test from the HC population was independently performed 1000 times to determine whether the proportion of the subgroup reached that of the RELN-del group. A resampling test using RELN-del data as a seed was also performed using the same procedure. *P*-values <0.05 were considered statistically significant.

## Supplementary information

Supplementary figures.

Supplementary Movie 1.

Supplementary Movie 2.
