# Supplementary material for: Cell body shape and directional movement stability in human-induced pluripotent stem cell-derived dopaminergic neurons
Source: Sci Rep. 2020 Apr 2;10:5820. doi: 10.1038/s41598-020-62598-4 (PMC7118143; doi:10.1038/s41598-020-62598-4)
Supplement: Supplementary file 1 — Supplementary figures. [file 41598_2020_62598_MOESM1_ESM.doc]

**Cell body shape and directional movement stability in human-induced pluripotent stem cell-derived dopaminergic neurons**

Yuko Arioka^1, 2, 3^, †^,*^, Emiko Shishido^1, 4^, †, Itaru Kushima^1, 5^, Daisuke Mori^1, 6^, Norio Ozaki^1^

^1^ Department of Psychiatry, Nagoya University Graduate School of Medicine, Nagoya, Japan

^2^ Center for Advanced Medicine and Clinical Research, Nagoya University Hospital, Nagoya, Japan

^3^ Institute for Advanced Research, Nagoya University, Nagoya, Japan

^4^ National Institute for Physiological Sciences, Okazaki, Japan

^5^ Medical Genomics Center, Nagoya University Hospital, Nagoya, Japan

^6^ Brain and Mind Research Center, Nagoya University, Nagoya, Japan

† These authors contributed equally to this work

*Corresponding author

Yuko Arioka, PhD

Center for Advanced Medicine and Clinical Research,

Nagoya University Hospital, Nagoya, 466-8560, Japan

Tel: +81 52 744 2942; Fax: +81 52 744 1303

Email: [ariokay@med.nagoya-u.ac.jp](mailto:ariokay@med.nagoya-u.ac.jp)

**
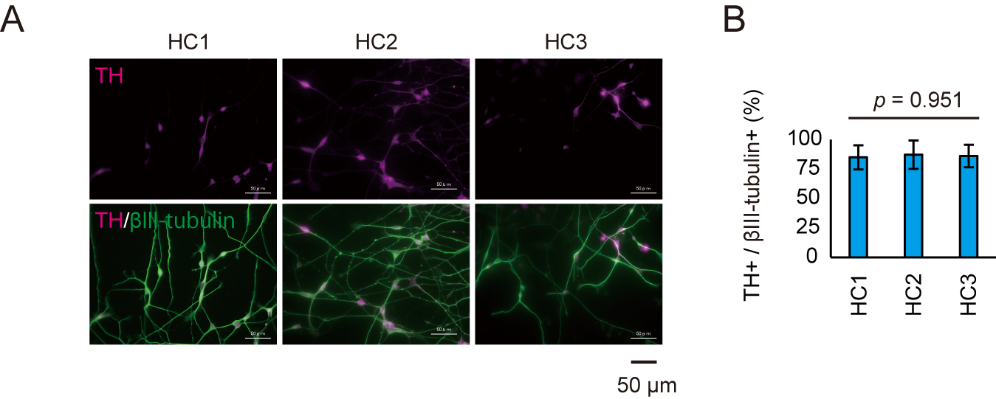
**

**Supplementary Figure 1. *iPSC-derived dopaminergic neurons***

(**A**) Representative images of immunostaining for TH and βIII-tubulin at 48 h after plating neurospheres. (**B**) Analysis of dopaminergic neuron differentiation efficiency by quantifying the ratio of TH^+^ to βIII-tubulin^+^ cells. Five fields were used. Bars represent mean ± SD.

**
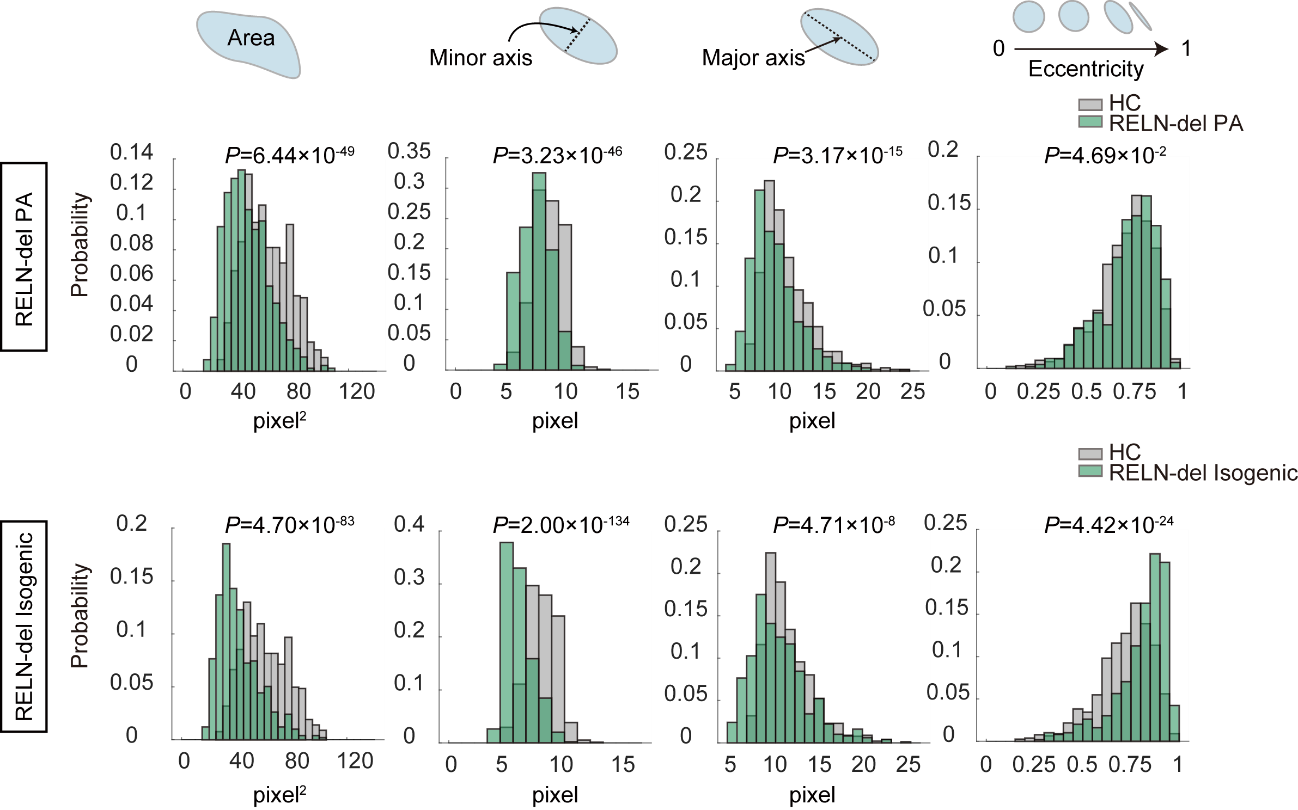
**

**Supplementary Figure 2. *Cell shape* *of RELN-del PA and RELN-del isogenic cells. (Associated with Fig. 2D)***

Comparisons of areas, lengths of the minor/major axes of cell bodies, and eccentricity of fitted ellipses of bodies of HC (gray) and RELN-del (green) cells. RELN-del PA (n = 535) and RELN-del isogenic (n = 497) cells show similar results.

**
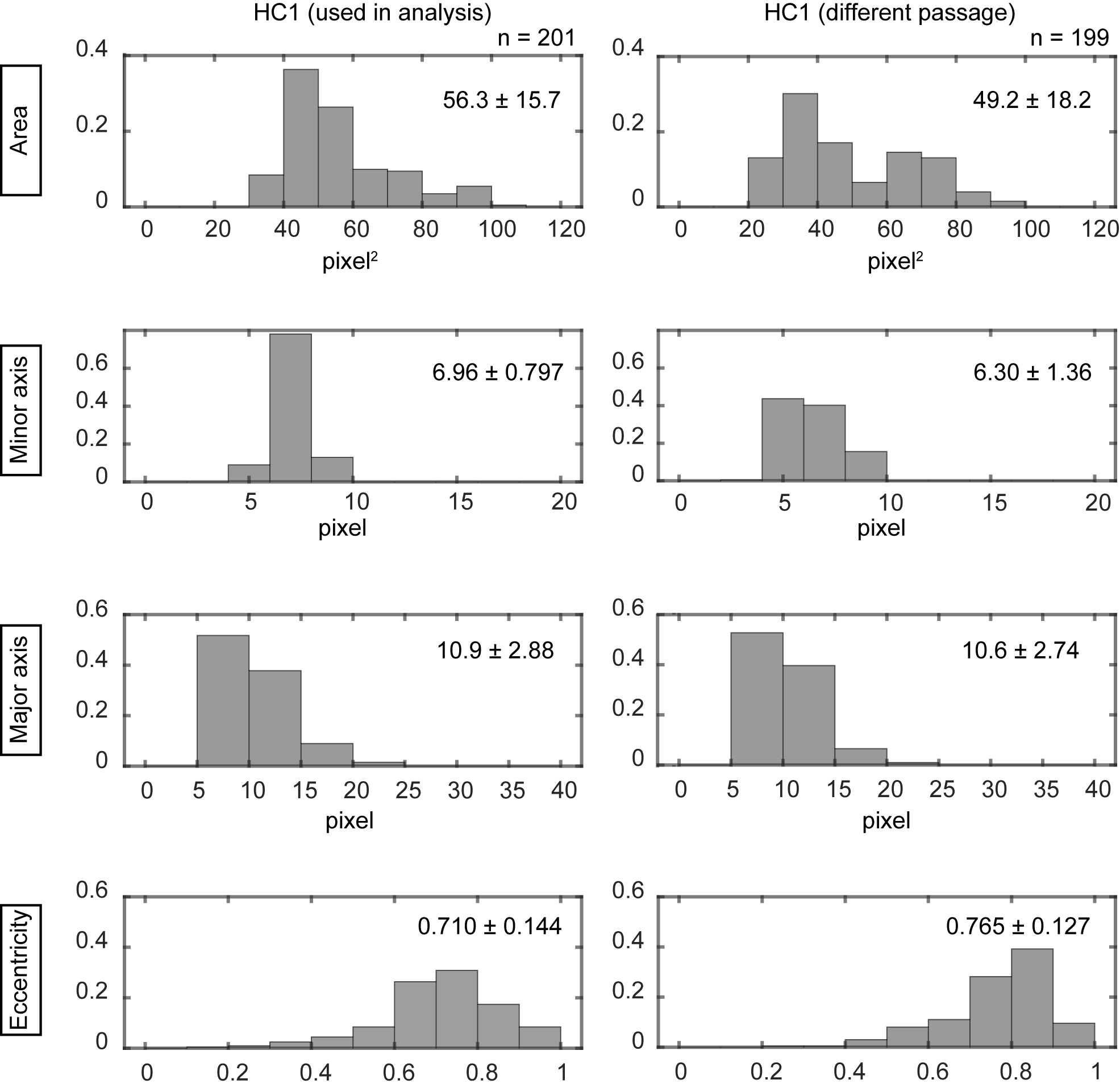

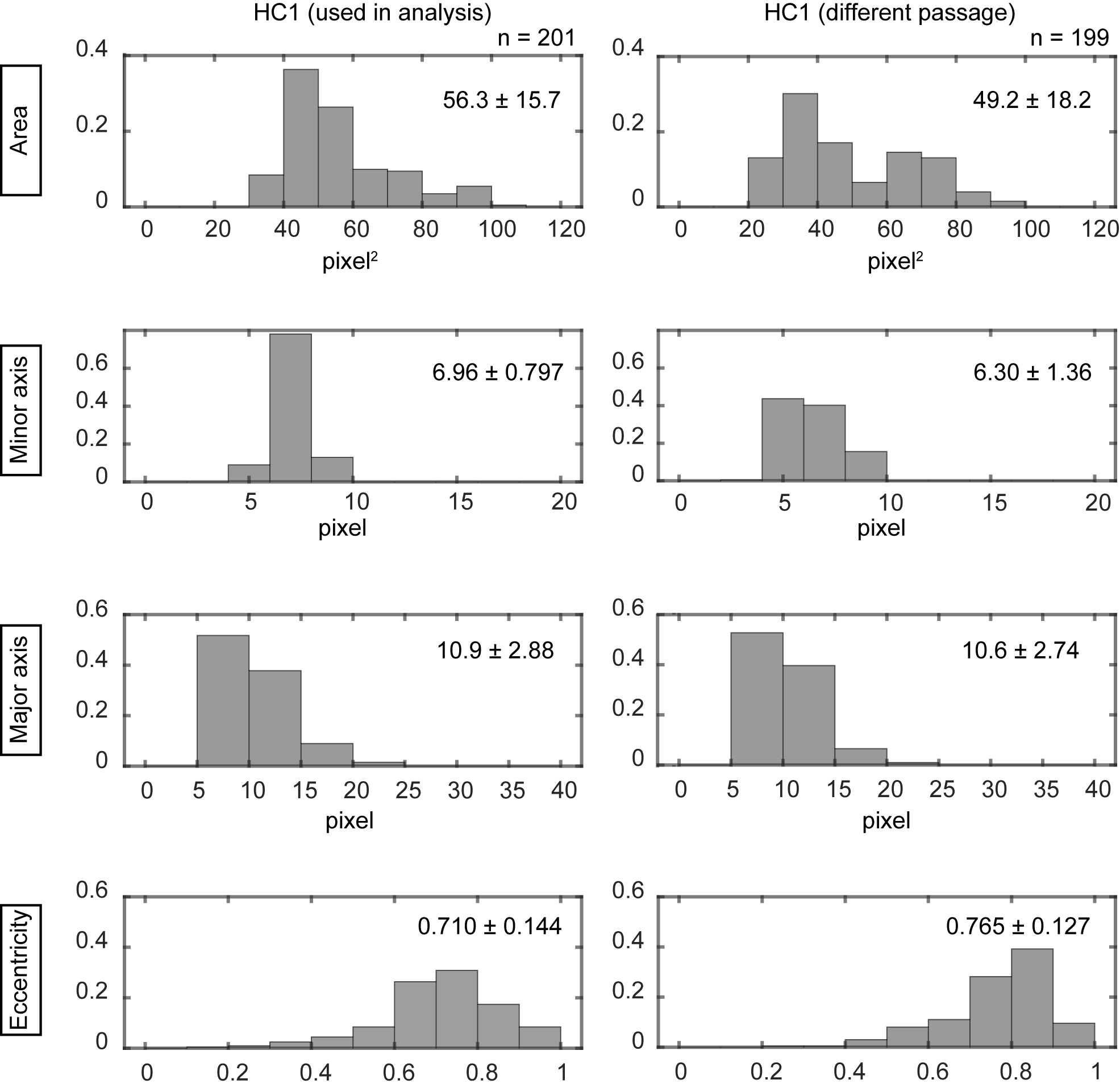
**

**Supplementary Figure 3. *Comparison of cells at different passages***

HC1 iPS cells were compared at different passage numbers. Numbers in boxes indicate means ± SD for each. *P-*values from analysis *vs.* different passages for Area, 3.93 × 10^−5^; Minor axis, 4.44 × 10^−9^; Major axis, 2.80 × 10^−1^; Eccentricity, 8.13 × 10^−5^.


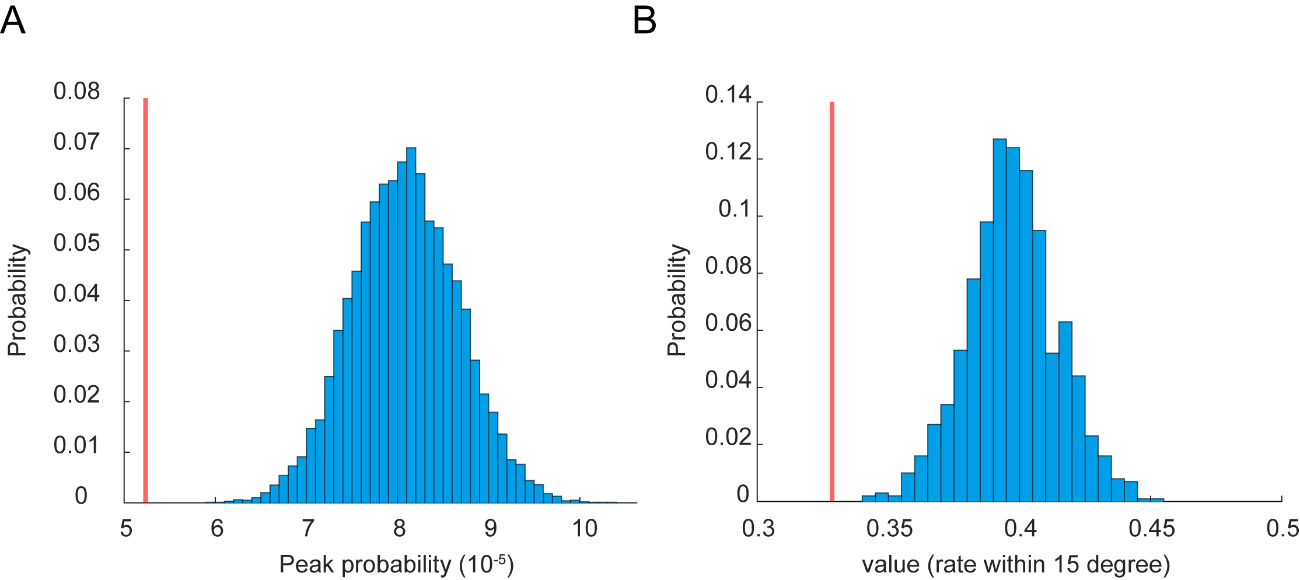


**Supplementary Figure 4. *Random sampling test***

Results of random sampling using HC data as a seed. (**A**) Random sampling test for bivariate angular data presented in Fig. 4C. The y-axis indicates the probability of the normalized number of trials. After 10000 trials, no case showed peak probability lower than the RELN-del cell peak probability (red vertical line, peak probability = 5.2 × 10^−5^). (**B**) Random sampling test for angular differences between movement vector and cell major axis is shown in Fig 5D. The y-axis indicates the probability of the normalized number of trials. After 1000 trials, no case showed a subpopulation lower than the RELN-del cell ratio (red vertical line, probability = 0.328).

**
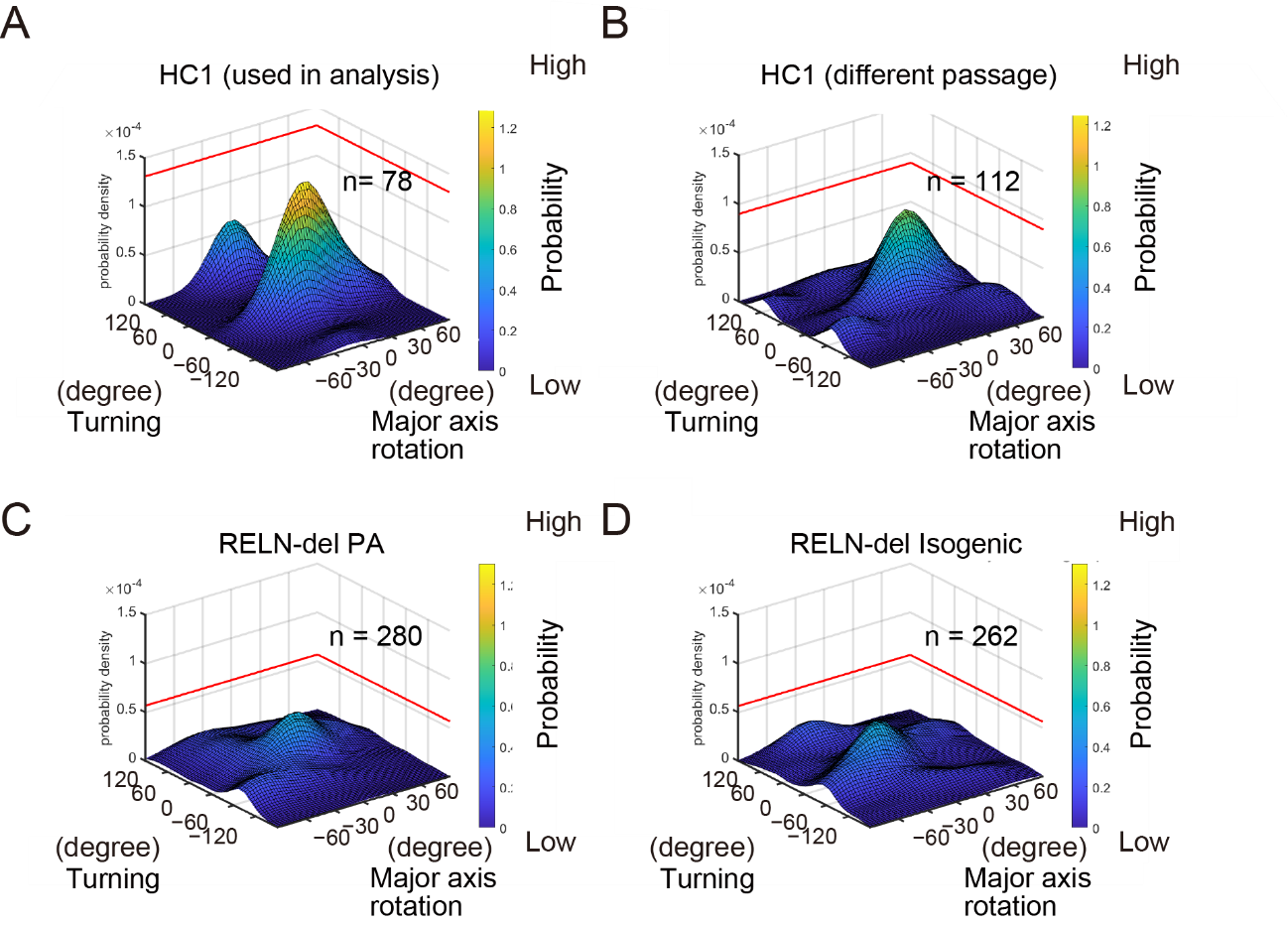
**

**Supplementary Figure 5. *Cell axis rotation and turning direction of subsets of HC and RELN cells***

Data were processed same as in Figs. 3 and 4 but with different subsets for (1) evaluation of the effect of different passages, and (2) comparing RELN-del PA and RELN-del isogenic cell lines. (**A**, **B**) Turning directions and cell axis rotations were plotted as divalent data for two HC1 subsets with different passage numbers. **A** is from the experimental data used in the present study (Figs. 2, 4, and 5) and the data in **B** represents iPSCs at different passage numbers. The associated data distributions with the kernel smoother were different (peak probability, A = 1.3 × 10^−4^, B = 9.0 × 10^−5^) but both had single peaks in the center. (**C, D)** Subsets of RELN-del lines; the peak probability of **C** was 5.7 × 10^−5^ and that of **D** was 5.6 × 10^−5^. The difference between **A** and **B** is smaller than the differences between **A** and **C** or **A** and **D**.


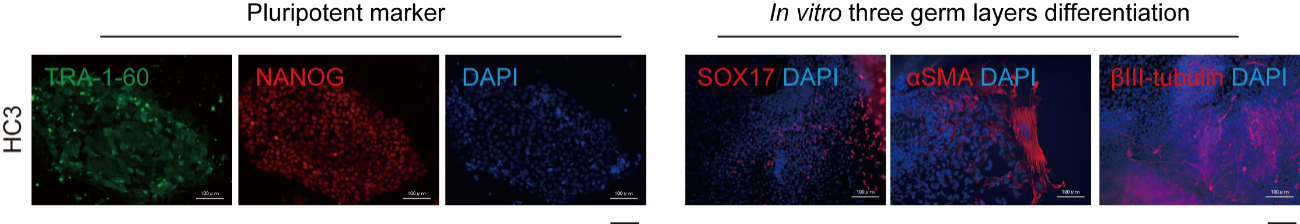


**Supplementary Figure 6. *Generation of iPS cells***

Left panel: Immunostaining for TRA-1-60 and NANOG in iPSC lines. Right panel: Evaluation of the capacity to differentiate into all three germ layers by SOX17 staining (endodermal marker), αSMA staining (mesodermal marker), and βIII-tubulin staining (ectodermal marker). The black scale bar under the images represents 100 μm.

**
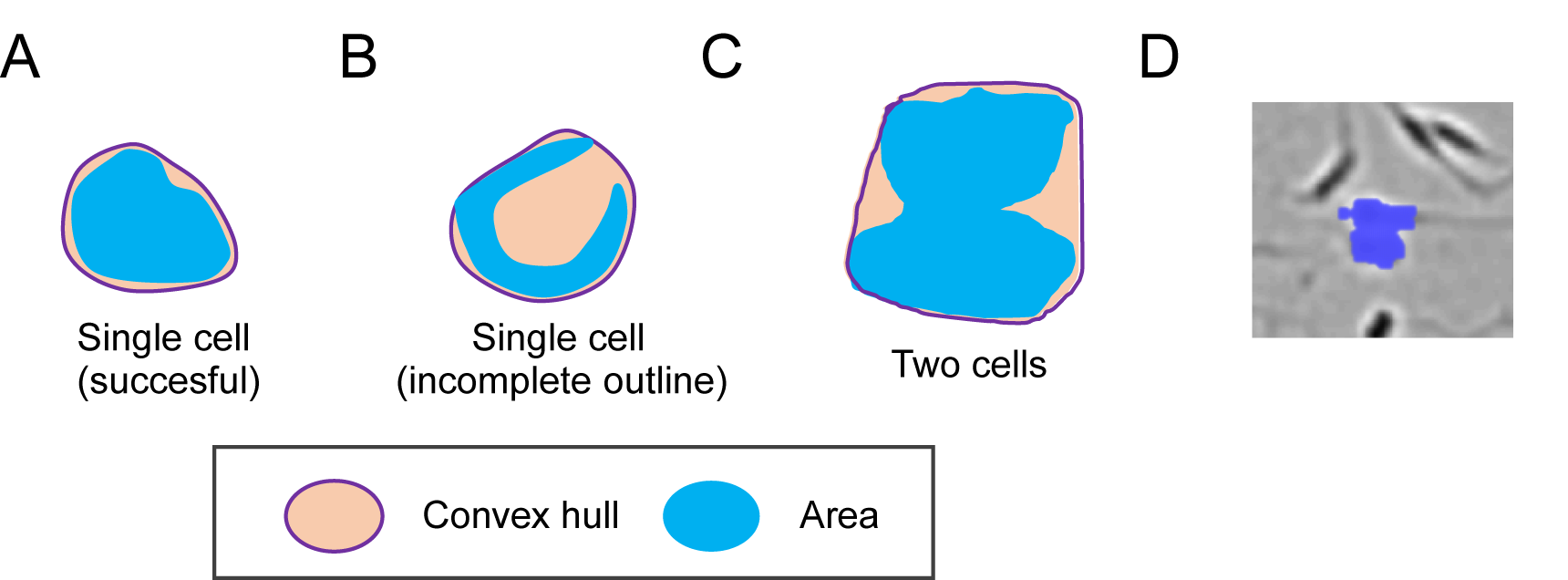
**

**Supplementary Figure 7. *Use of convex hull for exclusion of misidentified cases***

Exclusion algorithm using a convex hull; (**A**) the successfully identified single cell has an area with a round shape. If a convex hull is applied to the area, it is almost the same as that of the area, such that the area and convex hull have similar sizes. (**B**) A representative type of misidentified case; when the closing step of image analysis is incomplete, the area has a crescent shape and the pixel size of the convex hull is much larger than that of the area. (**C**) Another type of misidentified case; when two cells are close to each other, they are detected as one area and the convex hull becomes bigger than the area. We excluded such cases in **B** and **C** by examining ratios of area and convex hull sizes. (**D**) Example of raw image frame of **C**.

**Supplementary Movies 1 and 2. *Automated detection of the cell body region during migration***

The area of the cell body region was shadowed blue and automatically assigned by image segmentation of phase-contrast images. Movie 1 shows cells from HC1, and Movie 2 shows cells from RELN-del PA.
